# Exogenous LEA proteins expression enhances cold tolerance in mammalian cells by reducing oxidative stress

**DOI:** 10.1038/s41598-025-86499-6

**Published:** 2025-01-27

**Authors:** Martina Lo Sterzo, Domenico Iuso, Luca Palazzese, Margherita Moncada, Francesca Boffa, Aurora Scudieri, Luisa Gioia, Marta Czernik, Pasqualino Loi

**Affiliations:** 1 Department of Veterinary Medicine, University of Teramo, Via Renato Balzarini 1, 64100 Teramo, Italy; 2 Department of Bioscience and Technology for Food, Agriculture and Environment, University of Teramo, Via Renato Balzarini 1, 64100 Teramo, Italy; 3 Institute of Genetics and Animal Biotechnology, Polish Academy of Sciences, Jastrzebiec, 05-552 Warsaw, Poland

**Keywords:** Late Embryogenesis Abundant proteins, Cells, Cryoprotection, Antioxidants, Biotechnology, Cell biology

## Abstract

Understanding the molecular mechanisms that confer cold resistance in mammalian cells might be relevant for advancing medical applications. This study aimed to exploit the protective function of Late Embryogenesis Abundant (LEA) proteins, known to provide resistance to low temperatures in extremophiles and plants, by their exogenous expression in mammalian cells, and compare their effects with the well characterized antioxidant, vitamin E.

Remarkably, the expression of LEA proteins in mammalian cells exerted cold-protective effect similar to Vitamin E. LEA proteins preserved cell viability during cold stress and ensured a normal metabolic activity after warming. Their protective action was due to the mitigation of cold-induced mitochondrial stress and the overproduction of reactive oxygen species (ROS), leading in turn to enhanced cytoskeleton stability and decreased DNA damage. Our studies showed that plant-derived LEA proteins exhibit remarkable cold protection effects in mammalian cells through their potent antioxidant properties, which was found comparable to Vitamin E.

**Supplementary Information:**

The online version contains supplementary material available at 10.1038/s41598-025-86499-6.

## Introduction

The capability to survive and adapt to environmental thermal stress appears to be a fundamental requirement for cell survival, representing a widespread mechanism among a wide range of organisms, from bacteria to plants and animals.

While heat shock response has been extensively studied in a variety of model cell systems and involves the upregulation of highly conserved proteins known as heat shock proteins (HSPs)^1^, cell cold-stress response is less described. This is quite surprising, because this is relevant not only for the general understanding of the molecular and biological mechanisms underlying cell resilience, but even for its potential application in temporary storage of cells, organs and tissues, and for therapeutic treatments^2^.

In mammals cold stress has different and severe biological effects, including reduction in translation/transduction, cell cycle arrest, slowed metabolism, change in membrane fluidity and cytoskeletal dynamics^3^. In particular, low temperatures can alter the membrane potential of mitochondria, leading to changes in their structure and function, ultimately increasing oxidative stress^4^. Cold adaptation in mammals is explored mainly through the study of hibernators as an experimental model^5^. However, a literature survey reveals that plants^6^, bacteria^7^, and yeast^3^ respond to, and cope with low temperatures, as well other extreme environmental conditions including drought, high salinity, heat and oxidation, by entering a state of metabolic dormancy^8^. These organisms maintain a more-or-less constant degree of membrane fluidity (homeoviscous adaptation)^9^ to counteract the stiffness of the cell membrane at lower temperature. Besides membrane protection, an induced synthesis of osmotically active, such as the low-molecular weight disaccharide trehalose and other sugars^10,11^, various protein classes, including cold-shock proteins (CSPs)^12^ or Late Embryogenesis Abundant (LEA) proteins^13^, accumulate in the intracellular compartment for osmotic balance, exerting on the same time a “bulk” protection of the overall cell structure. Despite being firstly discovered to accumulate in the late stages of embryo development in cotton seeds^14^, LEA proteins have been later documented in a broad range of organisms, like in bacteria^15^, fungi^16^, nematodes^17^, rotifers^18^, embryos of the brine shrimp *Artemia franciscana*^19^, the arctic springtail *Megaphorura arctica*^20^, the chironomid larva *Polypedilum vanderplanki*^21^, and tardigrades^22^.

The exact molecular function of LEA proteins is still unclear. They are classified as intrinsically disordered proteins^23^, due to their high hydrophilicity and amino acid composition responsible for their unfolded conformation in solution. Changes in the environmental condition induces their folding into secondary and tertiary structure^24^ that allow their interaction and stabilization of target proteins/enzymes, preventing their denaturation and subsequent aggregation when exposed to low water availability, like desiccation^25^, or other stressful condition, such as extreme temperatures^26,27^. Furthermore, available data about the mechanism of action of LEA proteins suggest that they act as stabilizers, hydration buffers, membrane protectants^28^, antioxidants, organic glass formers, and/or ion chelators^29,30^.

Here, we examined the effects of two LEA proteins on embryonic sheep fibroblasts exposed to cold stress: the cold acclimation protein WCOR410 from *Triticum aestivum*, that targets the plasma membrane, and the dehydrin-1dhn RAB17 from *Zea mays*, found in the nucleo-cytoplasm.

We found that LEA-induced expression in fibroblasts kept a low temperature showed cold-protection effects overlapping those exerted by Vitamin E. This allowed us to identify cellular pathways linking LEA proteins with resistance to mitochondria-mediated oxidative stress, reduced DNA damage, mitochondria and cytoskeleton stability in the cold.

These findings suggest that LEA proteins mimic the antioxidant action of Vitamin E, highlighting the potential of LEA proteins as valuable tools for introducing cold-resistance strategies into mammalian systems and the effectiveness of Vitamin E as a positive control for such studies.

## Results

### Subcellular localization of exogenous-expressed LEA proteins in sheep fibroblasts

**Fig. 1:**
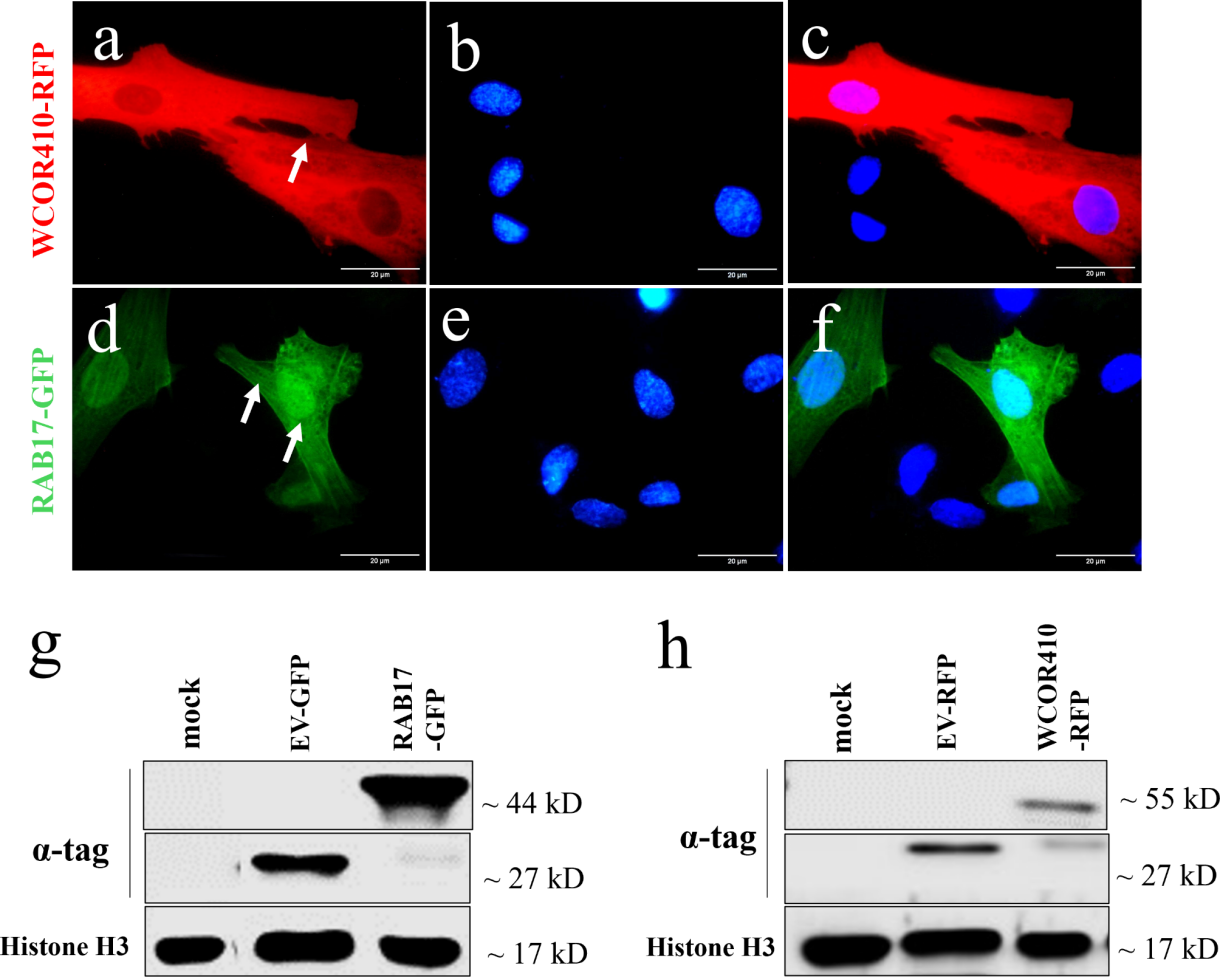
Subcellular localization of exogenous-expressed LEA proteins in sheep fibroblasts. (**a**–**c**) pTag-WCOR410-RFP; (**d**–**f**) pTag-RAB17-GFP. (**a**) WCOR410-RFP fusion protein displays membrane localization (arrowheads), (**b**) nuclei stained with Hoechst 33342; (**c**) merged; (**d**) RAB17-GFP fusion protein displays cytoplasmic and nuclear localisation (arrowheads); (**e**) nuclei stained with Hoechst 33342; (**f**) merged. (**g**) Western blot verified expression of pTag-RAB17-GFP in the somatic cells; (**h**) Western blot verified expression of pTag-WCOR410-RFP in the somatic cells. Protein extract from non-transfected cells (mock) and transfected with empty vectors EV-GFP and EV-RFP was used as a control. Scale bars, 20 μm (**a**–**f**).

Sheep embryonic fibroblasts were transiently transfected with pTag-WCOR410-RFP and pTag-RAB17-GFP. Subcellular localization of each LEA protein is shown on Fig. 1. WCOR410-RFP protein showed a peri-membrane localization (Fig. 1a), while RAB17-GFP was located in the cytoplasm and nucleus of the cells (Fig. 1d). As a control, sheep fibroblasts were transfected with empty vectors, pTags-RFP-N and pTags-GFP-N. Unlike LEA-positive cells, the results showed that GFP and RFP tag exhibited a widespread distribution throughout the cells (Supplementary Data 1). Western blotting analysis was used to confirm LEA proteins expression in sheep fibroblasts, as well as only fluorescent tags (RFP, GFP) as a control (Fig. 1g, h).

### Improved cold-resistance of sheep embryonic fibroblast expressing LEA proteins

Sheep embryonic fibroblasts transfected with pTag-WCOR410-RFP and pTag-RAB17-GFP, as well as non-transfected control (CTR), were exposed to 10 °C and 4 °C for one week.

**Fig. 2:**
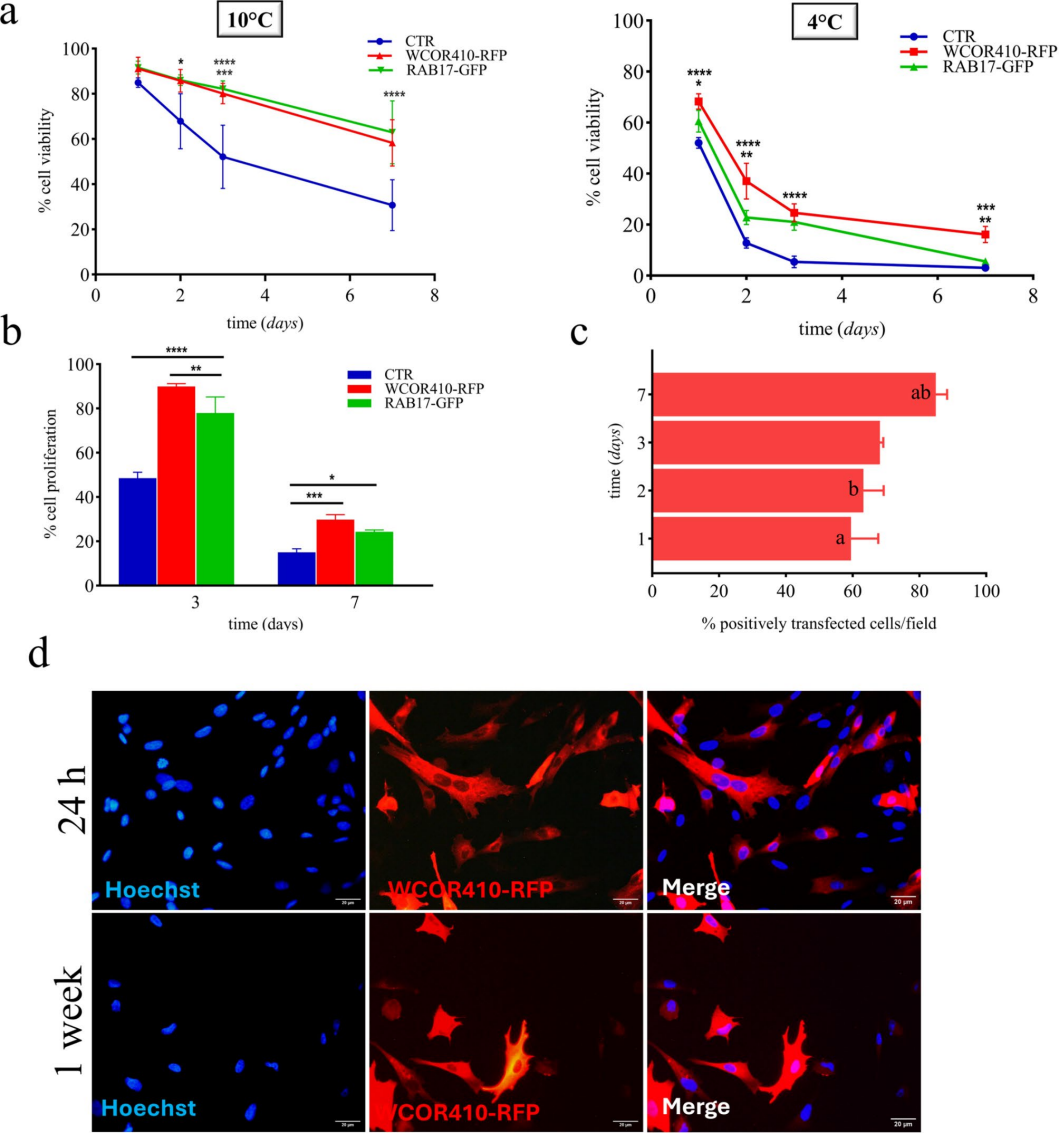
LEA proteins enhance cell metabolic activity and viability of sheep fibroblasts exposed to cold stress. (**a**) Left: cell viability of sheep fibroblasts transfected with single LEA protein exposed to 10 °C for up to 7 days. Viability of the cell with Trypan Blue was controlled after 1-, 2-, 3- and 7 days (2-Way Anova plus post hoc Tukey test for multiple LEA groups and control at different time of cold-exposure; *p < 0.05 for CTR vs. WCOR410 and RAB17 (2 days); ***p < 0.001 for CTR vs. WCOR410 (3 days); ****p < 0.0001 for CTR vs. RAB17 (3 days); for CTR vs. WCOR410 and RAB17 (7 days). Error bars indicate SD. Right: cell viability of sheep fibroblasts transfected with single LEA protein exposed to 4 °C for up to 7 days. Viability of the cell with Trypan Blue was controlled after 1-, 2-, 3- and 7 days (2-Way Anova plus post hoc Tukey test for multiple LEA groups and control at different time of cold-exposure; *p < 0.05 for CTR vs. RAB17 (1 day) and WCOR410 vs. RAB17 (1 day); **p < 0.01 for CTR vs. RAB17 (2 days) and WCOR410 vs. RAB17 (7 days); ***p < 0.001 for CTR vs. WCOR410 (7 days); ****p < 0.0001 for CTR vs. WCOR410 (1-, 2-, 3 days) and RAB17 (3 days) and for WCOR410 vs. RAB17 (3 days)). Error bars indicate SD. (**b**) Percentage of metabolic active LEA-transfected cells after 3- and 7 days of cold stress. Cellular metabolic activity was assessed with MTT assay after cells were put back in culture for 24 h (2-Way Anova plus post hoc Tukey test for multiple LEA groups and control at different time of cold-exposure; *p < 0.05 for CTR vs. RAB17 (7 days); **p < 0.01 for WCOR410 vs. RAB17 (3 days); ***p < 0.001 for CTR vs. WCOR410 (7 days); ****p < 0.0001 for CTR vs. WCOR410 and RAB17 (3 days)). Error bars indicate SD. (**c**) Percentage of LEA-positive cells per field remained attached to culture dish after 1-, 2-, 3- and 7 days of cold stress (one-Way Anova plus post hoc Tukey test for LEA group at different time of cold-exposure; same letter indicate statistical difference between groups; *p < 0.05 for 1 vs. 7 days and for 2 vs. 7 days). Error bars indicate SD. (**d**) Representative fluorescence image of WCOR410-RFP positive cells per field after 1 day (upper) and 7 days (lower). Scale bar, 20 μm.

After 1-, 2-, 3- and 7 days, cell viability was assessed using the Trypan Blue exclusion test. The results, shown in Fig. 2a, left, demonstrated that after 1 day of cold treatment at 10 °C no statistical difference in cell viability was detected between groups. After 2- and 3 days the control group began to differ significantly from both LEA proteins-expressing cells. In particular, the percentage of viable cells was 85.8% ± 5.02, 86.1% ± 2.36, and 67.8% ± 12.17 for WCOR410, RAB17 and CTR respectively after 2 days. After 3 days, cell viability decreased to 80.2% ± 4.54, 82.2% ± 3.51 and 52.2% ± 13.97 for WCOR410, RAB17 and CTR, respectively. After one week, only 30.7% ± 11.27 of cells are viable in the control group, while expressed LEA proteins were able to protect sheep embryonic fibroblasts, as indicated by their significantly higher cell viability in both LEA’s groups (WCOR410: 58.3% ± 10.26; RAB17: 63% ± 13.93).

When comparing cold treatment at 4 °C, we observed that cell viability decreased much more rapidly in each group (Fig. 2a, right), halving within 24 h and nearly reaching zero after 3 days in control cells (52.1% ± 2.11 in 1 day; 12.8% ± 2 in 2 days; 5.3% ± 2.3 in 3 days; 3.1% ± 1.4 in 7 days). In contrast, in LEA-transfected cells, although the rapid decline in viability followed the same trend, we observed a statistically significant higher viability compared to controls (WCOR410: 68.3% ± 3 in 1 day; 37% ± 7 in 2 days; 24.6% ± 3.5 in 3 days; 16.1% ± 3.15 in 7 days) (RAB17: 60.5% ± 4.27 in 1 day; 22.8% ± 2.75 in 2 days; 21.05% ± 3.26 in 3 days; 5.5% ± 1.4 in 7 days).

Furthermore, a cell metabolic activity assay using mitochondrial-dependent reduction of 3-[4,5-dimethylthiazol-2-yl]-2,5-diphenyl tetrazolium bromide (MTT assay) was performed. This test allows the determination of metabolically active cells, focusing on enzymatic activities, especially those associated with mitochondria. Results indicated that LEA-transfected cells revealed a higher metabolic activity compared to controls following a rescue assay after cells cold-exposure for 3- and 7 days (Fig. 2b).

Moreover, the results highlighted that LEA-positive cells were significantly more resilient to cold exposure compared to non-expressing cells. This observation is supported by the fact that over time, the percentage of LEA-positive cells per field progressively increased from 59.5% ± 8.19 after 1 day to 84.9% ± 3.36 after 7 days of cold-stress (Fig. 2c, d), demonstrating their improved capability to recover from cold stress remaining alive and attached to the culture dish.

### Improved cold-resistance of sheep fibroblast treated with α-tocopherol (Vitamin E)

**Fig. 3:**
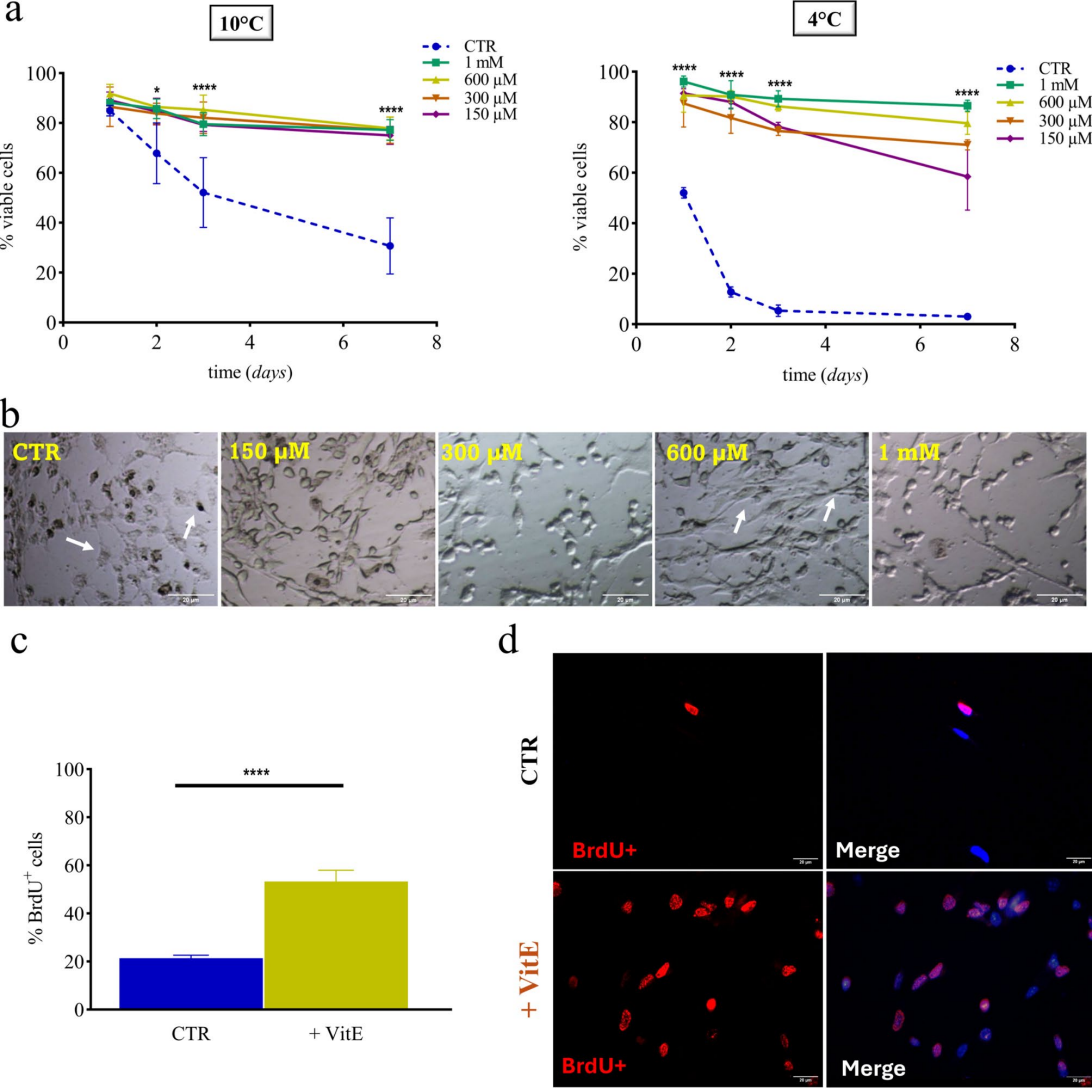
Sheep fibroblasts treated with α-Tocopherol (Vitamin E) during cold stress enhance their proliferation and viability. (**a**) Left: cell viability of sheep fibroblasts treated with different concentration of Vitamin E exposed to 10 °C for up to 7 days. Viability of cells with Trypan Blue was assessed after 1-, 2-, 3- and 7 days (2-Way Anova plus post hoc Tukey test for multiple Vitamin E concentration groups and control at different time of cold-exposure; *p < 0.05 for CTR vs. 1 mM, 600 µM, 300 µM, 150 µM (2 days); ****p < 0.0001 for CTR vs. 1 mM, 600 µM, 300 µM, 150 µM (3 and 7 days)). Error bars indicate SD. Right: cell viability of sheep fibroblasts treated with different concentrations of Vitamin E exposed to 4 °C for up to 7 days. Viability of cells with Trypan Blue was controlled after 1-, 2-, 3- and 7 days (2-Way Anova plus post hoc Tukey test for multiple Vitamin E concentration groups and control at different time of cold-exposure; ****p < 0.0001 for CTR vs. 1 mM, 600 µM, 300 µM, 150 µM (1-, 2-, 3- and 7 days). Error bars indicate SD. (**b**) Sheep fibroblasts treated with Vitamin E during cold stress imaged with a phase contrast inverted microscope. Pictures were captured after culture of cells treated with different concentrations of Vitamin E (1 mM, 600 µM, 300 µM, 150 µM) and cold-exposed for 3 days. Scale bar, 20 μm. (**c**) Percentage of proliferating Vitamin E-treated cells after cold stress. Cells were treated with 600 µM Vitamin E and cold-exposed for 24 h. Proliferation was assessed after cold-stress treatment by counting the number of cells that had incorporated BrdU into the nuclei (Student’s t-test between control and 600 µM Vitamin E; ****p < 0.0001). Error bars indicate SD. (**d**) Fluorescence images of anti-BrdU assay of Vitamin E-treated cell (down pictures) and control (upper pictures) proliferation after cold exposure. Cells were treated with 600 µM Vitamin E and cold-exposed for 24 h together with not-treated cells as a control. Scale bar, 20 μm.

Sheep embryonic fibroblasts were further treated with different concentrations of α-Tocopherol (Vitamin E) ranging from 1 mM, 600 µM, 300 µM, to 150 µM, and then stored at 10 °C and 4 °C for up to one week. After 1-, 2-, 3- and 7 days cell viability was assessed using the Trypan Blue exclusion test. Results shown in Fig. 3a, left, demonstrated that at a temperature of 10 °C, cells treated with Vitamin E, regardless of concentration, followed the same trend as LEA protein-expressing cells making their protective effect comparable to control cells. Surprisingly, the results at 4 °C (Fig. 3a, right) showed that Vitamin E treatment not only exhibited concentration-dependent efficiency but also resulted in a marked increase in cell viability compared to LEA proteins. After just 24 h, the effect of Vitamin E proved to be decisive, showing a cell viability of around 90% for each concentration, compared to 52.1% ± 2.11 of control. After 3 days, when cell viability in control group dropped to 5.3% ± 2.3, cells treated with Vitamin E still exhibited a viability of 89.3% ± 3.2, 86.3% ± 1.8, 76.5% ± 1.7 and 78.3% ± 1.6, for 1 mM, 600 µM, 300 µM and 150 µM, respectively. After 7 days of cold exposure at 4 °C, cell viability decreased to 86.5% ± 2.2, 79.6% ± 4.4, 71% ± 2 and 58.5% ± 13.2, for 1 mM, 600 µM, 300 µM and 150 µM, respectively, compared to 3.1% ± 1.4 in the control.

Based on viability results, the concentration of 1 mM of Vitamin E was found to be the best. However, as shown on Fig. 3b (arrows), cells treated with a concentration of 600 µM instead of 1 mM exhibited a normal phenotype, with the typical fibroblast elongated shape, once returned in culture after cold stress. Lower concentrations of Vitamin E, like 300 µM to 150 µM, were no longer effective, as demonstrated by the cell viability data and the cell’s phenotype. Rounded and less proliferative phenotypes became even more evident in the control group, where all cells appeared dead, with a flattened appearance, a dark nucleus and a damaged cell membrane (Fig. 3b, arrows). Thus, the concentration of the Vitamin E of 600 µM was selected as the most effective for all subsequent experiments.

To further understand the effect of cold treatment we investigated cell proliferation after rescue using anti-BrdU fluorescence assay. BrdU (5-Bromo-2′-deoxyuridine) is a thymidine analogue that is incorporated into newly synthesized DNA strands when cells enter the S phase. Therefore, the amount of BrdU in the cell nucleus reflects the level of cell division and proliferation. The anti-BrdU assay was carried out after cold stress on cells treated with 600 µM of Vitamin E and control cells. The results obtained indicated that after cold exposure Vitamin E-treated cells revealed an higher proliferation rate compared to controls (Fig. 3c). The amount of BrdU in cell nuclei was measured using a fluorescent anti-BrdU conjugate (red colour) with cell nuclei counterstained by Hoechst 33342 (blue colour), as shown in Fig. 3d.

### Cold-induced mitochondrial network and cytoskeleton dysfunction

**Fig. 4:**
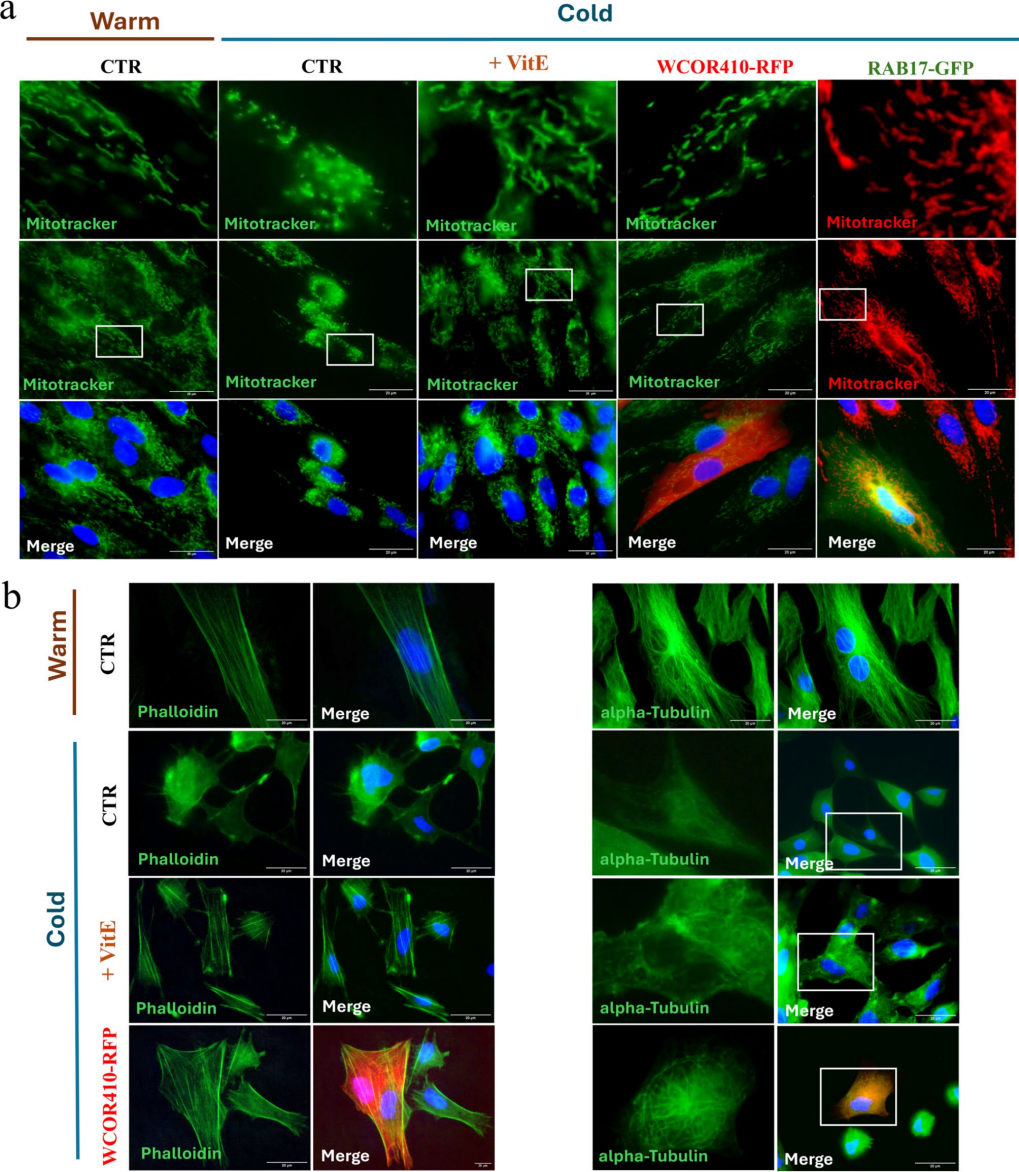
Differences in mitochondria and cytoskeleton cold stability. (**a**) Mitochondria staining performed using MitoTracker Green or MitoTracker Red dyes, revealing mitochondrial network morphology in cells cultured at 38.5 °C (CTR) and exposed to cold stress under different treatments (CTR, Vitamin E, WCOR410-RFP and RAB17-GFP). The nuclei were counterstained with Hoechst 33342. Scale bar, 20 μm. (**b**) Cytoskeleton staining performed using Phalloidin green to stain actin microfilaments (left panel) and tubulin immunofluorescence to stain microtubules (right panel) on cells cultured at 38.5 °C (CTR) and exposed to cold stress with different treatments (CTR, Vitamin E and WCOR410-RFP). Alpha-tubulin detected with Goat anti-Mouse IgG (H + L) Secondary Antibody, Alexa Fluor 488. Nuclei were counterstained with Hoechst 33342. Scale bar, 20 μm.

Sheep fibroblasts transfected with single LEA proteins, or treated with 600 µM Vitamin E, as well as non-transfected control, were cold stressed for 1 day. Normally cultured cells (38.5 °C) were used as a control. Results in Fig. 4a showed that both LEA proteins and Vitamin E protected the cellular mitochondrial network from cold stress. Cells expressing WCOR410-RFP, RAB17-GFP and Vitamin E did not show many cold-related mitochondrial damage, as indicated by MitoTracker green and red, highlighting a higher number of mitochondria with a normal, perinuclear distribution (Fig. 4a). Localisation and shape were similar to those obtained in control cells cultured at 38.5 °C. Differently, a massive mitochondria damage was observed in the control group after cold stress, where we noted a lower number of mitochondria, mostly peripherally located and fragmented without creating an elongated network.

We further analysed microfilaments and microtubules morphology using Phalloidin green staining or antibody against alpha-tubulin. At physiological temperatures (38.5 °C) sheep fibroblasts exhibited normal actin microfilaments spanning the entire cells (Fig. 4b, left panel) and long, organized microtubules (Fig. 4b, right panel). However, cold treatment led to cytoskeletal fragmentation and disorganization, resulting in the disappearance of most microfilaments and microtubules in control cells (Fig. 4b). In a marked contrast, cells expressing WCOR410-RFP and those treated with Vitamin E exhibited significantly less cold-related cytoskeleton damage. F-actin staining demonstrated the presence of normal actin microfilaments spanning the entire cells while, although alpha-tubulin immunostaining indicated some cytoskeletal disruption, the microtubules still remained more organized compared to the control cells (Fig. 4b).

### Cold induces overproduction of ROS

**Fig. 5:**
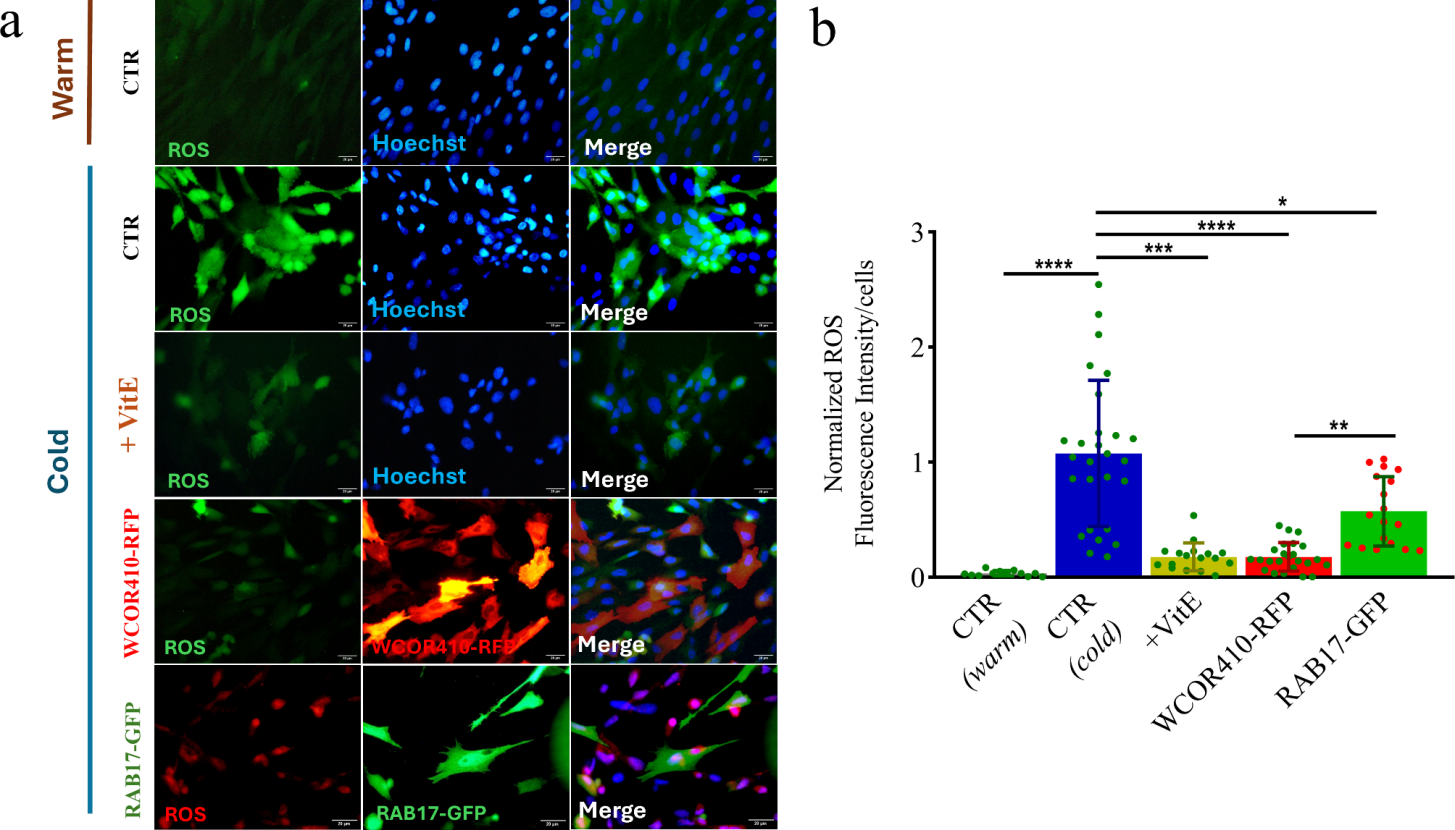
Oxidative stress in sheep fibroblasts caused by cold-induced mitochondria dysfunction. (**a**) ROS staining performed using H_2_DCFDA (green) and Cellular ROS red to assess oxidative stress in cells cultured at 38.5 °C (CTR) and exposed to cold stress under different treatments (CTR, Vitamin E, WCOR410-RFP and RAB17-GFP). The nuclei were counterstained with Hoechst 33342. Scale bar, 20 μm. (**b**) Quantification of ROS production on cells cultured at 38.5 °C and following cold exposure (Kruskal-Wallis plus post hoc Dunn test for multiple comparison; *p < 0.05 for CTR vs. RAB17; **p < 0.01 for WCOR410 vs. RAB17; ***p < 0.001 for CTR vs. Vitamin E; ****p < 0.0001 for CTR (warm) vs. CTR (cold) and CTR vs. WCOR410). Error bars indicate SD. Dot plots represent each analysed cell field.

Since mitochondrial dysfunction is usually correlated with ROS over-production^31,32^, we further evaluated the oxidative stress in cold-exposed cells. ROS level was measured as the oxidation rate of the cell-permeant fluorescent dye 2′,7′-dichlorodihydrofluorescein diacetate (H_2_DCFDA) or using the Cellular ROS Red Assay Kit, both sensitive to a wide range of ROS including hydrogen peroxide, singlet oxygen, superoxide anion, and hydroxyl radicals. ROS level was evaluated in LEA-transfected and Vitamin E-treated cells as well as in control group at the beginning (38.5 °C), and after cold treatment (Fig. 5a). We observed significantly higher cold-induced ROS production in control than in LEA-transfected cells (Fig. 5b), indicating that mitochondria response reflected the increased oxidative stress. Remarkably, when sheep fibroblasts were treated with Vitamin E during cold-stress, we observed a significant decrease in ROS production, to a level comparable to those of LEA-transfected cells exposed to cold (Fig. 5b).

### Cold-induced cell DNA damage

**Fig. 6:**
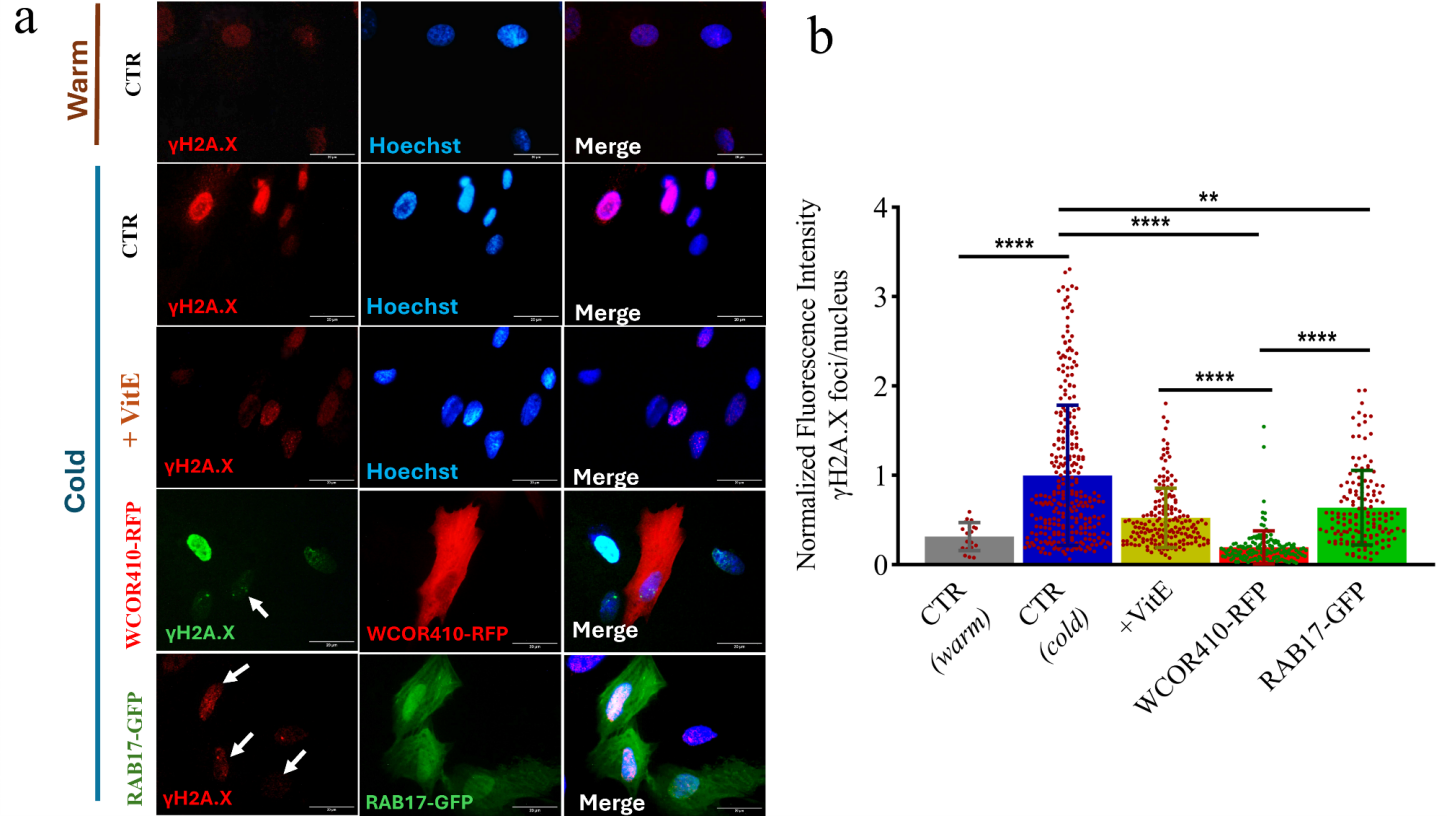
Induction of γH2A.X in sheep fibroblasts nuclei after cold-induced oxidative stress. (**a**) Immunofluorescence of γH2A.X in cells cultured at 38.5 °C (CTR) and exposed to cold stress under various treatments (CTR, Vitamin E, WCOR410-RFP, and RAB17-GFP) using Goat anti-Mouse IgG (H + L) secondary antibodies conjugated to Alexa Fluor 488 or Alexa Fluor 555. Nuclei were counterstained with Hoechst 33342. Scale bar, 20 μm. (**b**) Quantification of γH2A.X on cells cultured at 38.5 °C and following cold exposure (Kruskal-Wallis plus post hoc Dunn test for multiple comparison; **p < 0.01 for CTR vs. RAB17; ****p < 0.0001 for CTR (warm) vs. CTR (cold) and CTR vs. WCOR410 and Vitamin E, and for WCOR410 vs. RAB17 and Vitamin E). Error bars indicate SD. Dot plots represent individually analysed cell nuclei.

Because cell oxidative stress may oxidize and disrupt DNA, we hypothesized that LEA-positive cells possess higher DNA stability due to suppressed ROS production in response to cold treatment. To verify this hypothesis, we used immunofluorescence to assess the phosphorylation of the histone variant H2A.X induced by DNA double strand breaks (DSB) and thus a marker of DNA damage. Phosphorylated gamma H2A.X (**γ**H2A.X) plays a role in maintaining the DNA repair machinery and recruiting it to the DSB site^33^. DNA damage was evaluated in LEA-positive and Vitamin E-treated cells as well as in control group at the beginning (38.5 °C), and after cold treatment (Fig. 6a). We found a significant higher level of **γ**H2A.X nuclear foci in the control group compared to LEA-positive cells (Fig. 6b). Interestingly, the addition of Vitamin E during cold-treatment reduced the formation of **γ**H2A.X foci to levels similar to those observed in LEA-positive cells (Fig. 6b).

## Discussion

Here we have shown that LEA proteins significantly reduce ROS overproduction in mammalian cells exposed to a relatively long cold stress. In particular, our findings revealed that the expression of two LEA proteins (cold acclimation protein WCOR410 and dehydrin-1dhn RAB17) protect sheep fibroblasts from the negative effect of prolonged storage at low temperatures. We have observed that mainly positively transfected cells survived the cold stress conditions; in fact, the percentage of cells expressing LEA proteins per field increased over time. To partially explore their protection spectrum, we have compared two different low-temperatures: 10 °C and 4 °C, from 24 h up to one week. As expected, these cold temperatures induced a standby metabolic state in cells, preventing proliferation and resulting in cell death within one week for approximately 70% at 10 °C and 97% at 4 °C for control fibroblasts. In contrast, LEA-transfected cells significantly extended their dormancy and reduced cell death.

As expected, cell viability decreased much more rapidly at 4 °C, compared to 10 °C, even for LEA-transfected cells. However, in contrast to the control fibroblasts which exhibited complete cell death within 3 days at 4 °C, the LEA-transfected cells demonstrated a marked resilience, maintaining significantly higher viability levels, with more the 20% of the cells remaining viable after 3 days. Notably, at 4 °C cold acclimation protein WCOR410 conferred a significantly higher percentage of cell viability compared to dehydrin-1dhn RAB17. The distinctive internal sequences of WCOR410 suggest that its role may differ slightly from that of dehydrins^34^. WCOR410 is specifically upregulated during cold stress and accumulates significantly in freezing-tolerant wheat genotypes during cold acclimation, indicating a specialized function in enhancing freezing tolerance^35^. In particular, it has been reported that WCOR410 proteins accumulate near the plasma membrane of cells where freeze-induced dehydration is likely to be more severe, preventing membrane damage and destabilization^36^. In contrast, dehydrin-1dhn RAB17 has a broader function in responding to dehydration and ABA (abscisic acid)^37^, making it probably less specialized for cold-specific stress. This different protective efficiency suggest that the sequence and structural features of LEA proteins are critical factors. Future studies including animal-derived LEA proteins could explore whether other specific LEA protein, might yield better outcomes. This could provide deeper insights into their functional diversity and help optimize strategies to improve cold tolerance in mammalian cells.

A stress factor to be considered in our experimental conditions was hypoxia, likely consequence of having kept the cells for 7 days in a closed tube. However, at low temperatures, cellular metabolism slows down significantly, resulting in a drastic reduction in oxygen consumption^38^. Consequently, in ours experiments hypoxia may take longer to develop and may not become a primary issue immediately. We are confident that the pH did not chance in the tubes at the end of the experimental time, as it should happen in case of full metabolic activity of the cells, for not shifting in the culture media colour (Phenol Red in Hepes-buffered) was observed.

In our study, cellular metabolic activity assay, assessments of mitochondrial networks, as well as analyses of cytoskeleton under cold stress all jointly demonstrated the beneficial effects of LEA proteins. In LEA-expressing cells, mitochondria maintained a physiological elongated morphology at low temperatures. In contrast, in non-expressing cells, mitochondria became fragmented and rounded in most of the cells, which is characteristic of cold-induced mitochondrial fission^39^, coupled with ROS overproduction that anticipates the process of cold-induced apoptosis^40–42^. Additionally, a disorganized cytoskeleton after cold stress was detected in non-expressing cells, confirming that cold rapidly induce intracellular events leading to both actin^43^ and tubulin^44^ disassembly. Whereas LEA-positive cells maintained a physiological organization of both microfilaments and microtubules.

We delved further into the role of ROS and observed a notable reduction in their levels in LEA-transfected cells, suggesting a potential mechanism to counteract the oxidative stress. Our results confirmed that, beyond their established chaperone function of safeguarding proteins and cellular membrane structures, LEA proteins may also serve a physiological role as antioxidants, as already suggested^45,46^. Usually, cold stress may elevate cellular ion concentration^47^, increase ROS levels^5,48,49^ or altered the activities of key antioxidant defence system enzymes^50^. In this context, LEA proteins may mitigate oxidative stress through several mechanisms. They can directly scavenge ROS by selective sequestration of metal ions that generate ROS or can stabilize and protect crucial antioxidants enzymes in a chaperone-like manner, ensuring their correct activity^51^.

The excessive production of ROS in response to cold stress may cause DNA bases oxidation or DNA strand breakage due to free radical attacks on the DNA sugar-phosphate backbone^52^. Oxidative DNA damage induced by ROS includes mostly oxidized bases and single-strand breaks (SSBs)^53^. However, excessive production of ROS has been also associated with cooling-induced double-strand breaks (DSBs) formation^54^. A recent study reported the evidence that a significant fraction of DSBs depends on DNA oxidation, supporting the idea that excision of oxidized bases promotes SSBs next then converted to DSBs^55^.

Thus, DNA damage from various environmental stresses, including cold stress has been shown to increase intracellular level of ROS^56^, which in turns regulate apoptosis^57^. A novel pathway has been proposed in which DNA damage increases intracellular ROS levels through histone H2A.X^58^, which plays a crucial role in the DNA damage response (DDR) pathway^59^. In response to double strands breaks (DSBs) on DNA, histone H2A.X undergoes phosphorylation to form γH2A.X foci, which are essential for recruitment and maintenance of the DNA repair machinery at the DSB sites^33^. Indeed, in our experiments, control cells experiencing elevated ROS levels during cold stress exhibited significant cellular DNA damage, as evidenced by increased phosphorylation of histone H2A.X to form γH2A.X. In contrast, cells expressing LEA proteins did not show such elevated ROS levels or DNA damage, indicating their potent protective effect. Analogously, we observed that cell treatment with the antioxidant Vitamin E effectively reduced γH2A.X phosphorylation, supporting the hypothesis that DSBs can be directly linked to oxidative stress.

Altogether, this study highlights the importance of managing oxidative stress during cold-induced-metabolic standby and suggests that the induction of torpor in mammalian cells through antioxidants employment might enhance the protective mechanisms conferred by LEA proteins.

Notably, cold adaptation in plants^60^ or bacteria^61^ is facilitated by enhancing the antioxidant response. Similarly, genes involved in the response to oxidative stress are strongly upregulated during desiccation^62,63^, suggesting oxidative stress is a critical factor in inducing anhydrobiosis^64^.

Therefore, a robust antioxidant defence system, comprising both enzymatic (e.g. catalase, superoxide dismutase, glutathione peroxidase and glutathione reductase) and non-enzymatic (e.g. glutathione, ascorbic acid, carotenoids, tocopherol) antioxidants, is essential to ensure cellular survival during cold stress^65^ or desiccation^66^.

Analogously, hibernating mammals, which enter a state of dormancy, can overcome cellular damage at low temperature due to metabolic suppression, mitochondrial adaptation and antioxidant activity^67^. One of their extrinsic mechanisms of cold resistance in vivo relies on a tissue-specific increase of antioxidant enzyme protein expressions^68^ and increased metabolic levels of antioxidants such as Ascorbate (vitamin C)^69^ or α-Tocopherol^70,71^. α-Tocopherol, primarily acquired through the diet, plays a crucial role in inducing cold resistance, predominantly by preventing membrane lipid peroxidation^72^. Our initial approach using antioxidant Vitamin C did not yield significant results and was discontinued. However, treatment with Vitamin E during cellular cold stress significantly enhanced the survival and proliferation of sheep embryonic fibroblasts. This treatment improved mitochondrial network integrity, stabilized the cytoskeleton, and reduced DNA damage by mitigating oxidative stress, similar to the effects observed in LEA-positive cells. The ineffectiveness of vitamin C may be attributed to its primary action in aqueous environments, without associating to plasma membrane, which does not adequately protect against oxidative stress-induced damage to lipid membranes under cold temperatures. In contrast, the effect of the lipid-soluble antioxidant vitamin E to promote membrane repair bypassing the peroxidation of lipids, as reported in the study by Howard et al.^73^, supported our finding that vitamin E enhances cell protection during cold stress.

Furthermore, a crucial aspect of cold adaptation is the biochemical regulation of membrane bilayer fluidity at low temperatures, known as homeoviscous adaptation^9^. Tocopherol significantly influences membrane dynamic by maintaining its integrity and fluidity through its antioxidant properties, interactions with membrane lipids and protection of polyunsaturated fatty acids^74^.

Notably, our experiments revealed that Vitamin E treatment was even more effective at 4 °C compared to LEA proteins, whereas at 10 °C their effects were comparable. Probably, sheep fibroblasts temporarily preserved LEA protein expression for longer time at 10 °C. However, this rescue effect was not long observed at 4 °C treatment, perhaps due to cessation of LEA protein expression at lower temperature.

In conclusion, LEA proteins conferred protection against a relatively long cold stress mainly through antioxidant activity, by levering on physiological pathways operating in extremophilic organisms. The confirmation of LEA’s antioxidant effects and their ability to mimic the action of Vitamin E, underscores their potential to develop novel technologies to store cell and organ at low temperatures for biomedical applications like cold storage of organs prior surgical transplantation.

## Methods

### Reagents

All materials were purchased from Sigma Aldrich, Milan, unless otherwise stated.

### Primary culture

Sheep embryonic fibroblasts (SEF) were derived from sheep foetuses collected from local slaughterhouses as donated material for research. Primary cultures of SEF (between the fourth and the eight passages) were cultured in DMEM (Gibco, Milan, Italy) containing 2 mM glutamine, 26 mM NaHCO3, and 50 µg/ml gentamicin supplemented with 10% of Foetal Bovine Serum (FBS), in an incubator at 38.5 °C in a humidified atmosphere with 5% CO_2_ in air. Cells were detached using 0.25% trypsin-EDTA and cultured until 80% of confluency. When required during experimental procedures cells were counted using a haemocytometer.

### Construction of LEAp plasmids

Coding Sequences (CDS) for the LEA proteins were produced by gene synthesis (Dundee Cell Products, USA): WCOR410 (~ 0.8 kb; Triticum aestivum cold acclimation protein WCOR410; GenBank L29152.1), and RAB17 (~ 0.5 kb; Zea mays dehydrin-1dhn, GenBank NM_001111949.1). RAB17 and WCOR410 were subcloned into pET-15b (Novagen, Rome, Italy) under T7 promoter. Subsequently, CDS were amplified using AccuPrime Pfx DNA polymerase (Thermo Fisher), and inserted using EcoRI/HindIII into the pTag-GFP-N, pTag-RFP-N to obtain pRAB17-GFP and pWCOR410-RFP (all plasmid backbones were from Evrogen, Milan, Italy). Correct clones were confirmed by Sanger sequencing using ABI PRISM 3100 (Applied Biosystem).

### **Cell transfection**

3.0 × 10^5^ cells were plated in 35 mm dishes and cultured for 24 h in complete culture medium. After 24 h cells were transfected using TransIT-X2 kit (Mirus Bio, USA) according to the manufacturer’s protocol using 1 µg of pTag-WCOR410-RFP-N or pTag-RAB17-GFP-N individually. Additionally, empty vectors were used as a control: pTags-RFP-N and pTags-GFP-N. Then, cells were incubated at 38.5 °C in a humidified atmosphere of 5% CO_2_ in air. Transfection efficiency was evaluated after 48 h by chromatin counterstaining with 5 µg/mL of Hoechst 33342 for 10 min and then analysed on an epifluorescence microscope (Nikon, Eclipse E-800) to estimate the percentage of positive transfected cells. All experiments were performed 48 h post-transfection.

### LEA protein localization in somatic cells

Sheep embryonic fibroblasts transfected with pTag-WCOR410-RFP or pTag-RAB17-GFP individually, as well as empty vector controls, pTags-RFP-N and pTags-GFP-N were fixed with 4% paraformaldehyde (PFA) for 20 min at room temperature (RT). Cells were subsequently washed with PBS, and then counterstained with 5 µg/mL of Hoechst 33342 for 10 min. Then, cells were mounted on slides with Fluoromount Aqueous Mounting Medium and protein localization in LEA-positive cells was then evaluated using epifluorescence microscopy (Nikon, Eclipse E-800).

### Western blot

Proteins were isolated from sheep fibroblasts transfected with pTag-WCOR410-RFP, pTag-RAB17-GFP, pTags-RFP-N, pTags-GFP-N, as well as from the non-transfected cells (as a control). To obtain the proteins, cells were harvested with RIPA lysis buffer, supplemented with a protease inhibitor cocktail, then sonicated three times and centrifuged at 12,000 g for 15 min at 4 °C. The supernatant was collected into 1.5 mL Eppendorf tube and the protein content was determined using the BCA Protein Assay Kit (Thermo Fisher, Milan, Italy) according to the manufacture’s protocol. Cell lysates were mixed with Laemli sample buffer and heated at 60 °C for 10 min. An equal amount of protein (20 µg/lane) was loaded into 12% (wt/vol) sodium dodecyl sulphate–polyacrylamide gel for electrophoresis (SDS-PAGE), followed by transfer to a polyvinylidene difluoride (PVDF) membrane at 4 °C for 2 h at 200 mA.

After transfer, the PVDF membranes were blocked for 1 h with 5% Bovine Serum Albumin (BSA) at RT, and subsequently incubated overnight with rabbit anti-tagRFP (1:5000; Evrogen, Moscow, Russia; AB233) or rabbit anti-tag (CGY)FP (1:5000; Evrogen, Moscow, Russia; AB121) antibodies at 4 °C, overnight.

The next day, secondary antibodies goat anti-rabbit HRP-conjugate (1:10000; Santa Cruz Biotechnology, USA; sc-2030) were blotted for 1 h at RT. Detection was performed by Clarity Western ECL substrate (Bio-Rad, Hercules, CA, USA) as developed by Azure Biosystems c400 (Sierra Ct, Dublin, CA, USA).

### Cell cold-stress exposure and cell viability

Sheep embryonic fibroblasts transfected with pTag-WCOR410-RFP or pTag-RAB17-GFP individually were resuspended to 5.0 × 10^5^ cells/mL in complete culture medium supplemented with 50 mM Hepes. Additionally, the same number of non-transfected SEF were resuspended with complete culture medium supplemented with 50 mM Hepes and treated with different concentration of α-Tocopherol (1 mM, 600 µM, 300 µM, 150 µM) (Vitamin E; Sigma-Aldrich, T3251). Non transfected and non-treated fibroblasts were used as control. Then, 1 mL aliquots (medium + cells) were placed in 1.5 mL closed tube and stored at 10 °C or 4 °C for 1-, 2-, 3- and 7 days. After cold-stress exposure, cell viability was evaluated with a haemocytometer using Trypan Blue staining. Cell counting was repeated from 3 to 5 times for each experimental group at each time point. For both LEA-transfected groups, it was not possible to distinguish between LEA-positive and LEA-negative cells when using Trypan Blue for counting; therefore, the entire transfected population was counted.

### MTT and BrdU assays

After cold-stress exposure, cells transfected with pTag-WCOR410-RFP or pTag-RAB17-GFP, as well as non-transfected cells (as a control) were seeded into 96-well plates, at density of 1.5 × 10^4^ per well, and incubated overnight. Cell metabolic activity was assessed by the mitochondrial-dependent reduction of 3-[4,5-dimethylthiazol-2-yl]-2,5-diphenyl tetrazolium bromide (MTT; Sigma, St. Louis, MO, USA) to purple formazan. After 4 hours of incubation, the MTT solution was discarded and 100 µL of DMSO were added to dissolve the formazan crystals. Cells were analysed by Enspire Multimode Plate Reader (Perkin Elmer, MA, USA). The cell viability was calculated by subtracting the 630 nm OD background from the 570 nm OD total signal of cell-free blank of each sample and was expressed as the percentage of controls set to 100%.

For cells treated with Vitamin E, MTT assay was not recommended^75^. Therefore, to check how the cold exposure effect on the proliferation rate of SEF treated with Vitamin E, indirect immunocytochemistry of 5-Bromo-2′-deoxyuridine (BrdU) (11296736001, Roche, Swiss) was performed according to the manufacturer’s protocols. Untreated control cells were used as control.

Both MTT and BrdU assays were repeated 3 times. For BrdU at every repeat, images of 5 different fields were acquired using an epifluorescence microscope (Nikon, Eclipse E-800). The number of proliferative cells (BrdU positive) over total cell number was counted using Image J software. For MTT assay at every repeat, 5 different replicates per sample were analysed by Enspire Multimode Plate Reader. For both LEA-transfected groups, it was not possible to distinguish between LEA-positive and LEA-negative cells when performing MTT assay; therefore, the entire transfected population was analysed.

### Mitochondrial and cytoskeleton F-actin staining

For mitochondria and cytoskeleton F-actin staining, cells 48 h post-transfection, as well as non-transfected cells treated with Vitamin E and non-transfected control was kept directly in the fridge (as monolayer) for 24 h. After 1 day, cells were kept out of the fridge and immediately incubated with 1 µM of MitoTracker green FM or red (Invitrogen, Molecular Probes, Milan, Italy) for mitochondrial staining, or 1 µM of Phalloidin green (Sigma-Aldrich, P5282) for cytoskeleton F-actin staining. Phalloidin green staining required previous cell fixation in 4% paraformaldehyde (PFA) for 5 min at room temperature (RT) and subsequent permeabilization with 0.1% Triton X-100 in PBS. Then, cells from both staining were counterstained with 5 µg/mL of Hoechst 33342. Cells from each experimental group were analysed in three replicates under an epifluorescence microscope (Nikon, Eclipse E-800). Only LEA-positive cells were included in the analysis.

### Live cell imaging of ROS production

The amount of ROS produced after cell cold-stress exposure has been calculated by the oxidation rate of the fluorescent probe 2′,7′-dichlorodihydrofluorescein diacetate (H_2_DCFDA) (InVitrogen, Thermo Fischer Scientific, Waltham, USA; D399) or Cellular ROS Red Assay Kit (Abcam, Cambridge, UK; ab186027). To explore the influence of cold stress on ROS production, cells were either maintained in culture at 38.5 °C (as positive controls) or exposed to low temperature. Both LEA transfected and not-transfected cells as control and those treated with Vitamin E were cold-exposed as monolayer for 24 h. After that time, cells were immediately incubated with 5 µM of H_2_DCFDA or with Cellular ROS Red Assay Kit following the manufacturer’s protocol. Cells from each experimental group were analyzed in-vivo under an epifluorescence microscope (Nikon, Eclipse E-800). Experiments were repeated 3 times and five to seven different well-focused imaged areas from each experiment were measured. The fluorescence intensity per cell was measured after background subtraction using Image J software. For LEA-transfected group, the entire cell fields were analysed.

### γH2A.X and alpha-tubulin immunostaining

Immunostaining of γH2A.X and alpha-tubulin was conducted on LEA-positive cells, cells treated with Vitamin E as well as not-transfected/non-treated control, kept at low temperature for 24 h as monolayer. After 1 day, cells were immediately fixed with 4% paraformaldehyde (PFA) for 20 min at RT. Then cells were permeabilized with 0.5% (v/v) Triton X-100 in PBS for 10 min and transferred in blocking solution (10% BSA/0.1% Tween 20(v/v) in PBS) for 1 h (all at RT). Cells were then incubated with the primary mouse anti-p-Histone H2A.X or anti-alpha Tubulin antibody (1:50; Santa Cruz Biotechnology, USA, sc-517348; abcam, UK, ab7291) at 4 °C overnight in a humidified chamber. After 3 washes in blocking solution, the secondary antibody Goat anti-Mouse IgG (H + L) Cross-Adsorbed Secondary Antibody, Alexa Fluor 488 (1:500; InVitrogen, Thermo Fischer Scientific, Waltham, USA; A-11001) or Goat anti-Mouse IgG (H + L) Highly Cross-Adsorbed Secondary Antibody, Alexa Fluor 555 (1:500; InVitrogen, Thermo Fischer Scientific, Waltham, USA, A-21424) was incubated for 1 h at RT. The nuclei were counterstained with 5 µg/mL of Hoechst 33342. Images were captured using an epifluorescence microscope (Nikon, Eclipse E-800). Experiments were repeated at least 3 times. For γH2A.X detection assay at least 50 cell nuclei from each experiment were measured. Given the difficulty in counting the number of foci inside the nucleus because of the widespread pan-nuclear fluorescent signal^76^, we measured the fluorescence intensity of each nucleus setting the fluorescence intensity threshold to distinguish γH2A.X foci from background noise. The fluorescence intensity of foci per nucleus was measured after background subtraction using Image J software. For both γH2A.X and alpha-tubulin immunostaining analyses, only LEA-positive cells were included.

### Statistics and reproducibility

Two-way ANOVA plus post hoc Tukey test for multiple comparison were used to compare live cells and cell metabolic activity of each experimental group at different time point of cold treatment. One-way ANOVA plus post hoc Tukey test for multiple comparison were used to compare the percentage of positive-LEA cells at each time point. Kruskal–Wallis plus post hoc Dunn test for multiple comparison were used for ROS quantification and DNA damage data. Student’s *t* test was used to compare cell proliferation rate in Vitamin E-treated cell. Data reported in this paper are the mean (± SD) for each group. Statistical significance is defined as: *p < 0.05, **p < 0.01, ***p < 0.001, and ****p < 0.0001. Statistical analyses were performed using GraphPad Prism for Windows (Version 8.01, GraphPad Software, Inc, CA, USA).

## Electronic supplementary material

Below is the link to the electronic supplementary material.

Supplementary Material 1

Supplementary Material 2

## Data Availability

All experimental data supporting the results described in our work are available upon request to: Martina Lo Sterzo, email: mlosterzo@unite.it; or to Pasqualino Loi, email: ploi@unite.it.
