# Supplementary material for: Exogenous LEA proteins expression enhances cold tolerance in mammalian cells by reducing oxidative stress
Source: Sci Rep. 2025 Jan 27;15:3351. doi: 10.1038/s41598-025-86499-6 (PMC11772582; doi:10.1038/s41598-025-86499-6)
Supplement: Supplementary file 1 — Supplementary Material 1 [file 41598_2025_86499_MOESM1_ESM.docx]

**Supplementary Information**

**Exogenous LEA Protein Expression Enhances Cold Tolerance in Mammalian Cells by Reducing Oxidative Stress**

*Martina Lo Sterzo^1^, Domenico Iuso^1^, Luca Palazzese^1^, Margherita Moncada^1^, Francesca Boffa^1^, Aurora Scudieri^1^, Luisa Gioia^2^, Marta Czernik^1,3^, Pasqualino Loi^1*^*

Authors affiliation:

^1^ Department of Veterinary Medicine, University of Teramo, 64100, Teramo, Italy.

^2^ Department of Bioscience and Technology for Food, Agriculture and Environment, University of

Teramo, Via Renato Balzarini 1, 64100, Teramo, Italy.

^3^ Institute of Genetics and Animal Biotechnology of the Polish Academy of Sciences, Jastrzebiec, 05-552, Warsaw, Poland.

^*^Correspondence: Pasqualino Loi, Via Renato Balzarini 1, Campus Coste Sant’Agostino, University of Teramo, Teramo 64100, Italy; email: [ploi@unite.it](mailto:ploi@unite.it)


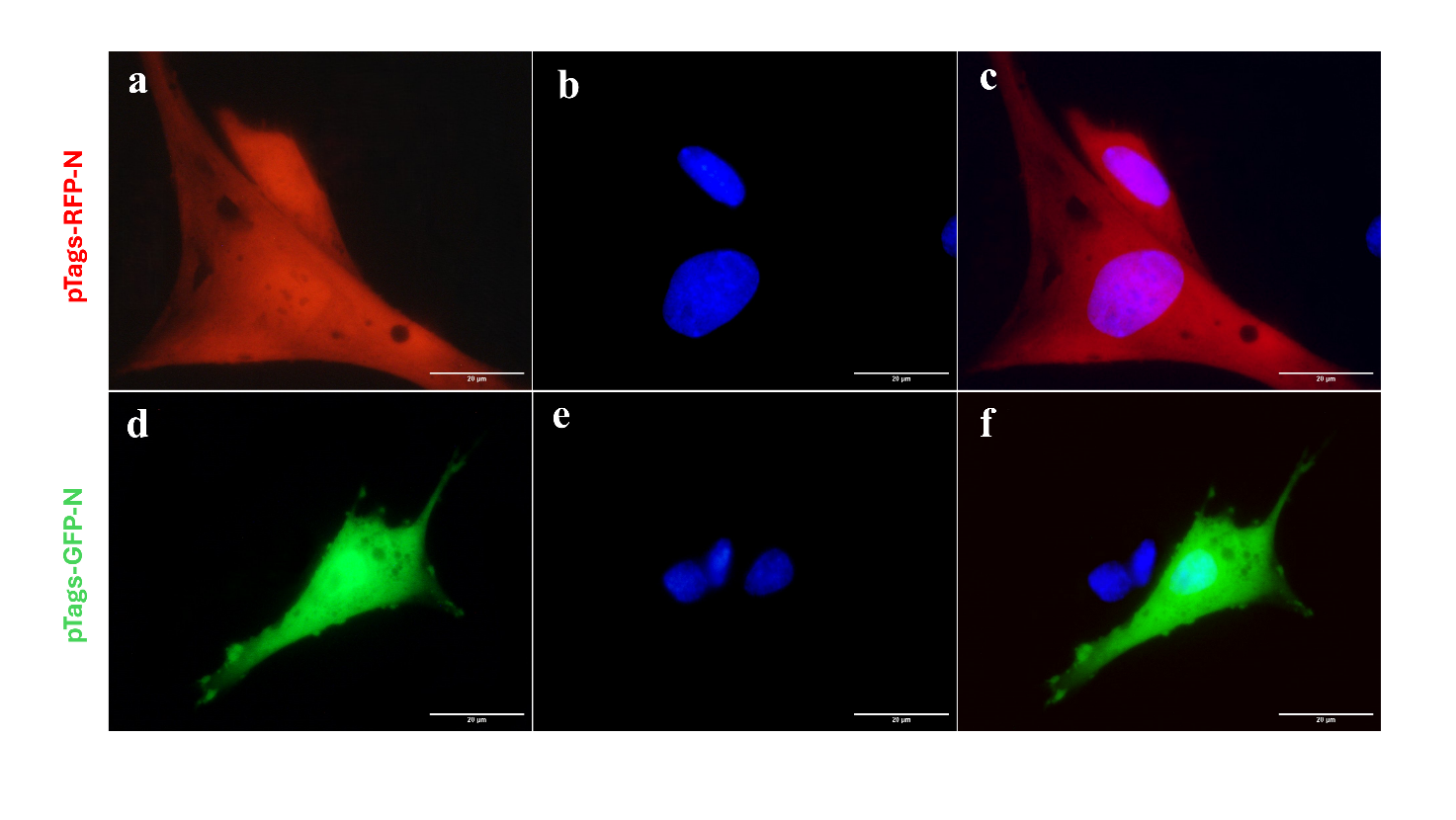


**Supplementary Data 1.**

As a control, sheep fibroblasts were transfected with empty vectors, pTags-RFP-N and pTags-GFP-N. GFP and RFP tags exhibited a widespread distribution throughout the cells, differently from LEA-positive cells.

**
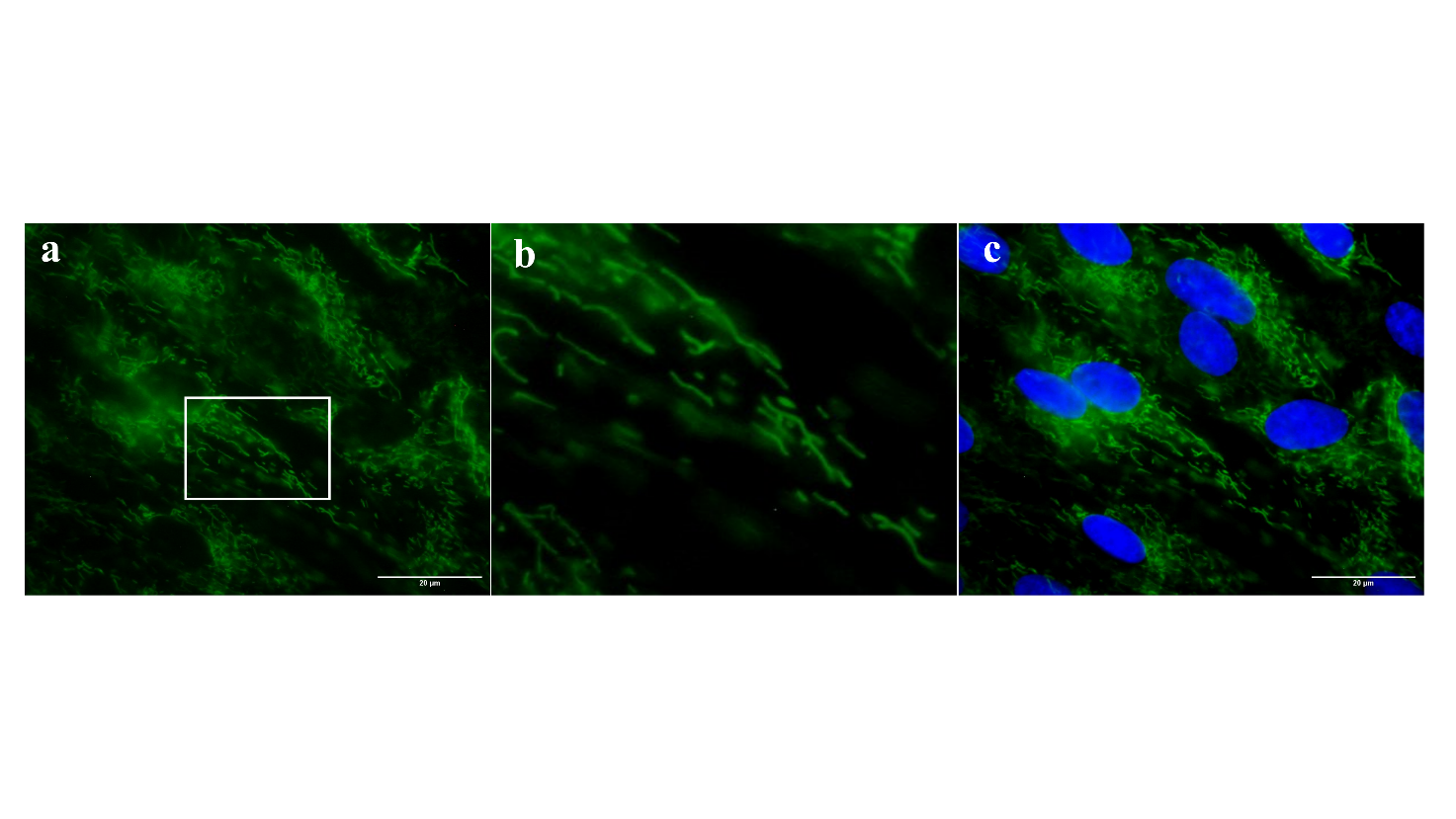
**

**Supplementary Data 2.**

As a positive control, sheep fibroblasts were normally cultured at 38.5°C and mitochondria were stained to compare their morphology from those exposed to cold stress. a) Mitochondria stained with Mitotracker green dye; (b) enlargement of a showing mitochondria form networks; (c) merged.

**
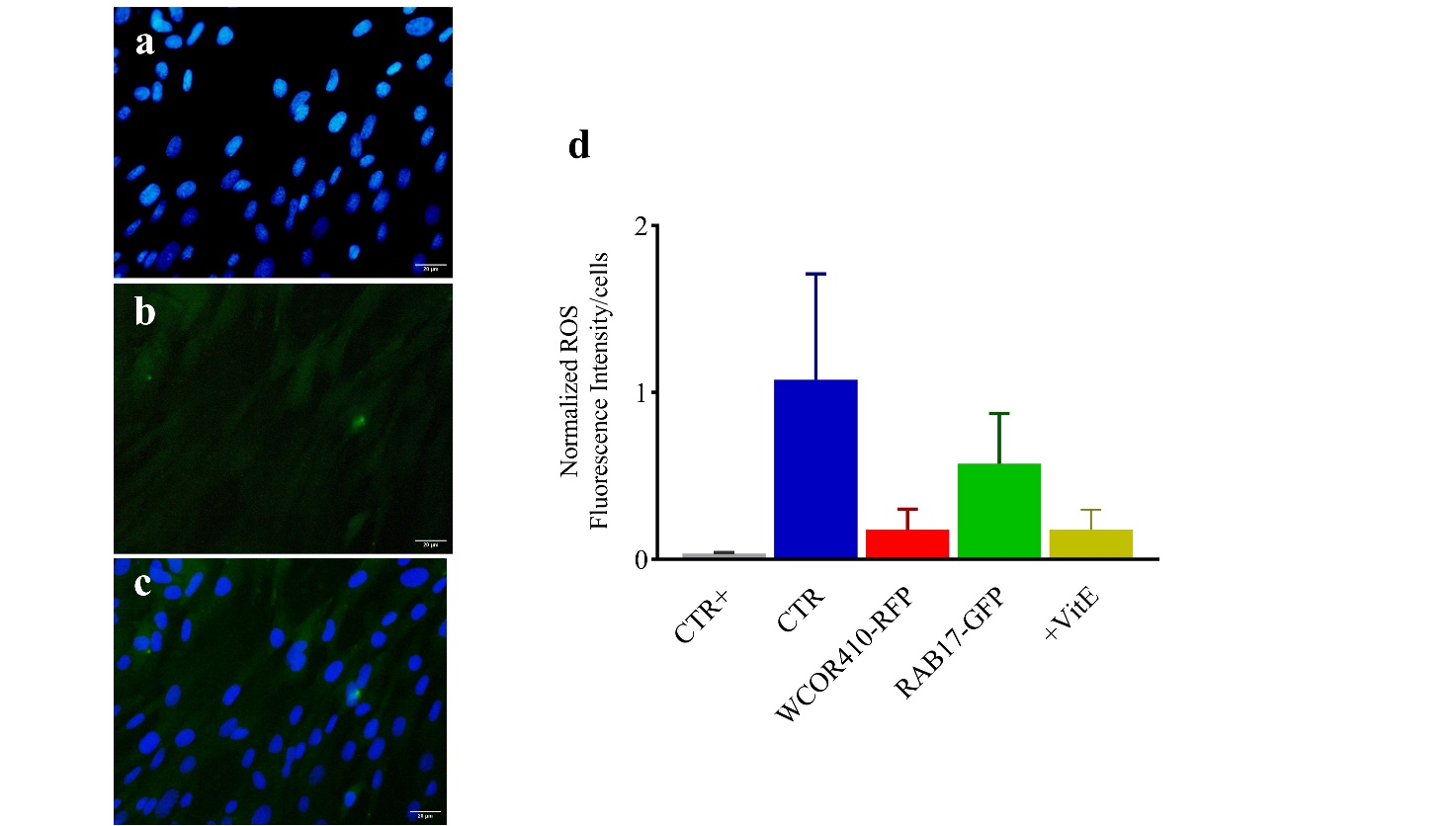
**

**Supplementary Data 3.**

As a positive control, sheep fibroblasts were normally cultured at 38.5°C and ROS probing were compared to those of cells exposed to cold stress. A weak fluorescent signal was observed in cell cultutred at 38.5°C, whereas cold-stressed cells exhibited a stronger fluorescence signal. This suggests that cold treatment generates ROS production within the cells. a) nuclei stained with Hoechst; (b) ROS probed with H_2_DCFDA; (c) merged; (d) Quantification of ROS production at 38.5 °C (CTR+) and following 24 h cold exposure (CTR, WCOR410-RFP, RAB17-GFP, Vitamin E)

**
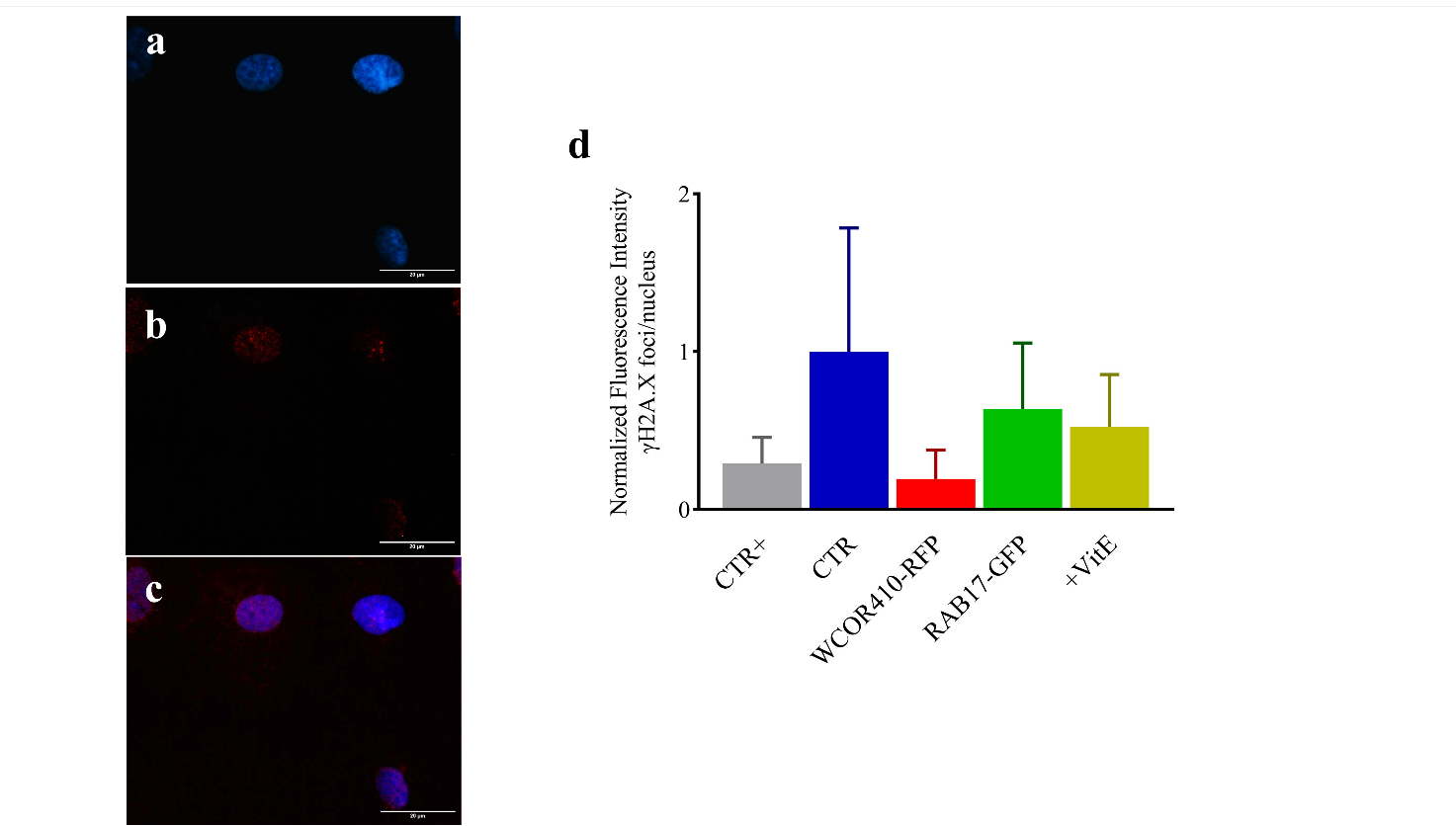
**

**Supplementary Data 4.**

As a positive control, sheep fibroblasts were normally cultured at 38.5°C and immunofluorescence of phosphorylated H2.AX (yh2ax) was used to compare DNA damage on normally cultured cells to those exposed to cold stress. (a) nucleus stained with Hoechst; b) γH2A.X detected with Goat anti-Mouse IgG (H+L) Secondary Antibody, Alexa Fluor™ 555; (c) merged; (d) Quantification of γH2A.X at 38.5 °C (CTR+) and following 24 h cold exposure (CTR, WCOR410-RFP, RAB17-GFP, Vitamin E).

**
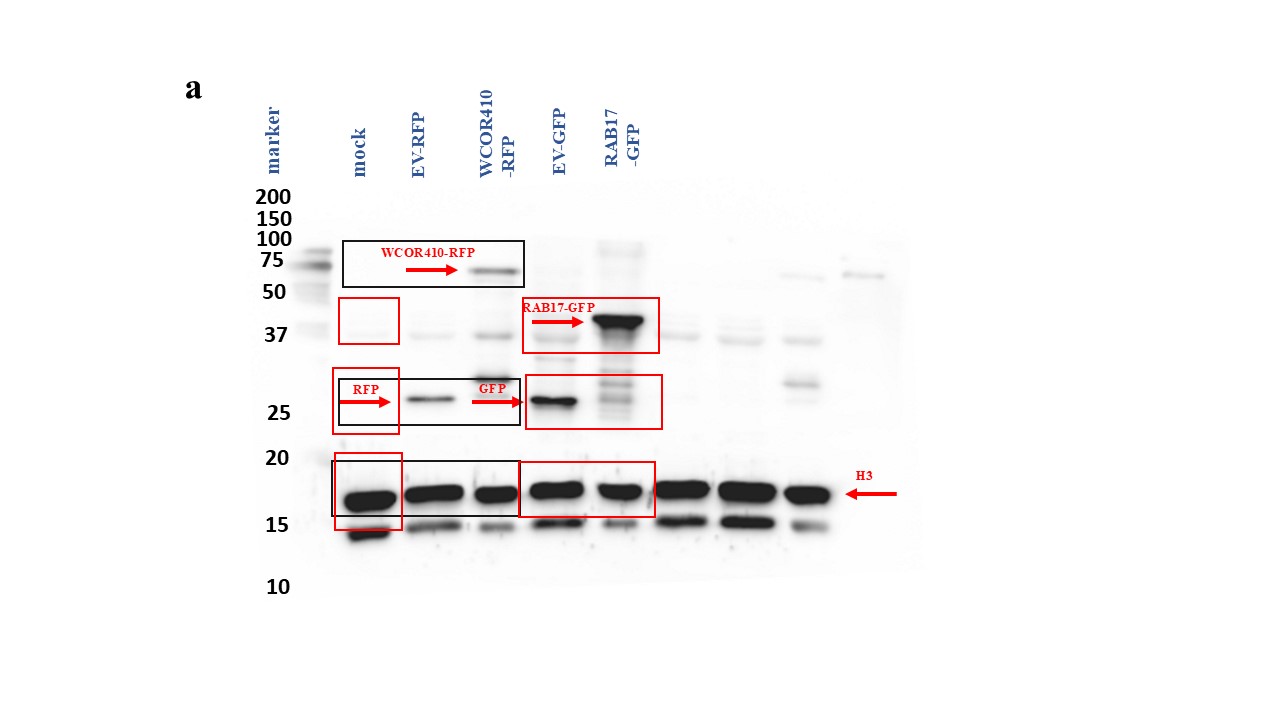
**

**Supplementary Data 5.**

Uncropped Western blot verified expression of pTag-RAB17-GFP and pTag-WCOR410-RFP in the somatic cells. Protein extract from non-transfected cells (mock) and transfected with empty vectors EV-GFP and EV-RFP was used as a control, membrane was blotted subsequently by anti-RFP and -GFP antibodies. Black square: figure 1h. Red square: figure 1g.
