# Supplementary material for: Exogenous LEA proteins expression enhances cold tolerance in mammalian cells by reducing oxidative stress
Source: Sci Rep. 2025 Jan 27;15:3351. doi: 10.1038/s41598-025-86499-6 (PMC11772582; doi:10.1038/s41598-025-86499-6)
Supplement: Supplementary file 2 — Supplementary Material 2 [file 41598_2025_86499_MOESM2_ESM.pdf]

Supplementary Information

Exogenous LEA Protein Expression Enhances Cold Tolerance in Mammalian Cells by Reducing Oxidative Stress

Martina Lo Sterzo[1], *Domenico Iuso*[1], *Luca Palazzese*[1], *Margherita Moncada*[1], *Francesca Boffa*[1], *Aurora Scudieri*[1], *Luisa Gioia*[2], *Marta Czernik*[1,3], *Pasqualino Loi*[1*]

Authors affiliation:

[1] Department of Veterinary Medicine, University of Teramo, 64100, Teramo, Italy.

[2] Department of Bioscience and Technology for Food, Agriculture and Environment, University of Teramo, Via Renato Balzarini 1, 64100, Teramo, Italy.

[3] Institute of Genetics and Animal Biotechnology of the Polish Academy of Sciences, Jastrzebiec, 05-552, Warsaw, Poland.

[*]Correspondence: Pasqualino Loi, Via Renato Balzarini 1, Campus Coste Sant'Agostino, University of Teramo, Teramo 64100, Italy; email: ploi@unite.it

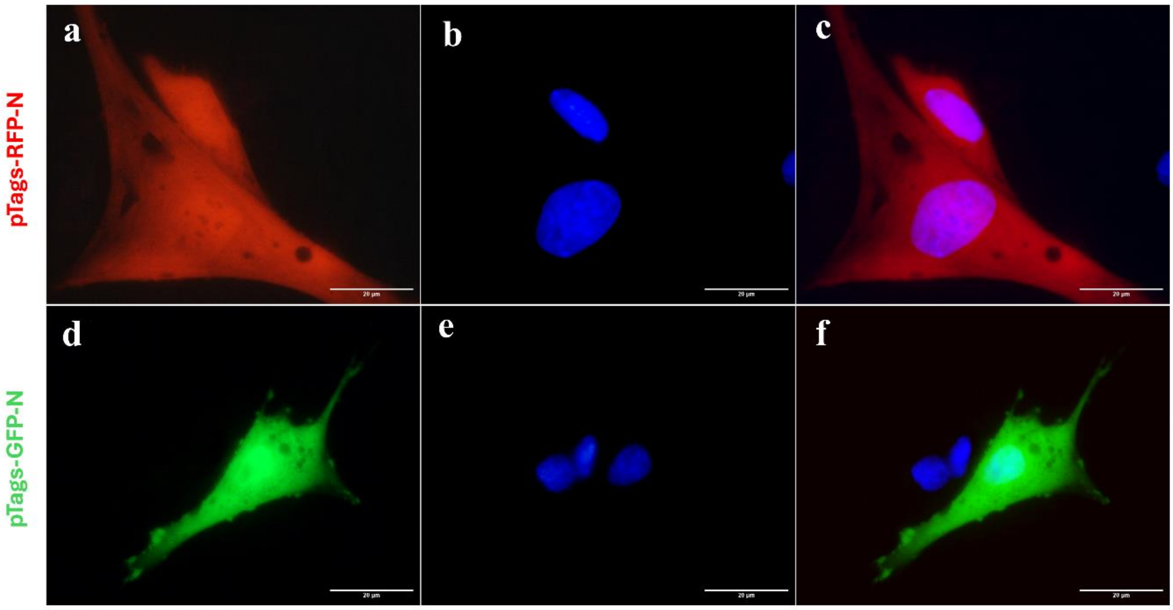

Supplementary Data 1.

As a control, sheep fibroblasts were transfected with empty vectors, pTags-RFP-N and pTags-GFP-N. GFP and RFP tags exhibited a widespread distribution throughout the cells, differently from LEA-positive cells.

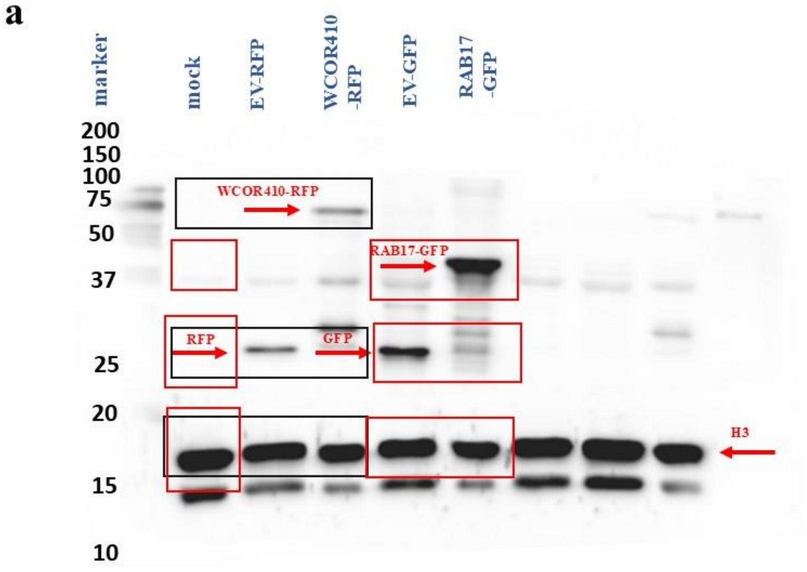

Supplementary Data 2.

Uncropped Western blot verified expression of pTag-RAB17-GFP and pTag-WCOR410-RFP in the somatic cells. Protein extract from non-transfected cells (mock) and transfected with empty vectors EV-GFP and EV-RFP was used as a control, membrane was blotted subsequently by anti-RFP and -GFP antibodies. Black square: figure 1h. Red square: figure 1g.
